# Response to comment about article ‘Systematic review and meta-analysis of goal-directed haemodynamic therapy algorithms during surgery for the prevention of surgical site infection’

**DOI:** 10.1016/j.eclinm.2025.103237

**Published:** 2025-05-03

**Authors:** Hasti Jalalzadeh, Rick H. Hulskes, Robert P. Weenink, Niels Wolfhagen, Ingeborg van Dusseldorp, Roald R. Schaad, Denise P. Veelo, Markus W. Hollmann, Marja A. Boermeester, Stijn W. de Jonge

**Affiliations:** a Department of Surgery, Amsterdam UMC, Location the University of Amsterdam, Amsterdam, the Netherlands; b Amsterdam Gastroenterology Endocrinology & Metabolism, Amsterdam, the Netherlands; c Dutch National Guideline Group for Prevention of Postoperative Surgical Site Infections, the Netherlands; d Department of Anaesthesiology, Amsterdam UMC, Location the University of Amsterdam, Amsterdam, the Netherlands; e Knowledge Institute for the Federation of Medical Specialists, Utrecht, the Netherlands

With great interest, we read the letter by Dr Hans Bahlmann, Dr Ingvar Halldestam and Dr Lena Nilsson regarding our publication, “Systematic review and meta-analysis of goal-directed haemodynamic therapy algorithms during surgery for the prevention of surgical site infection”.^1^ Indeed, the figure labels that indicated group allocation were reversed in Table 1: Meta-analysis of primary, secondary and subgroup analyses of the incidence of surgical site infection associated with goal-directed haemodynamic therapy, T3 Type of intervention: combination of fluids, vasopressors, and inotropes, and Digital Supplemental Appendix of Appendix 7E. Type of intervention: fluids, inotropes, and vasopressors. We are grateful for their attention to detail and for providing us the opportunity to correct this error but did not affect results and conclusions as analyses were done correctly.

The other concerns raised regarding supposed errors in data collected from one of the included trials do not result from errors but from differences in the definitions used.^2^ Part of the added value of a systematic review is that the authors organise all the reported information that can contribute to answering a specific question in a standard structure using common definitions. The standardisation may result in data handling that differs from that of the original study. In this case, there are two important differences in data handling.

## CDC definition of SSI

In accordance with the widely recognised CDC definition of SSI, we classified anastomotic leakage as organ/space SSI.^1^^,^^3^ Bahlmann and colleagues^2^ classified “anastomotic insufficiency”, defined as any leak requiring surgical intervention, as a separate outcome from SSI. To collect SSI data following the CDC definition, we had to combine these data. Accordingly, we scored 10 SSI in the intervention group (5 superficial wound infections/dehiscences + 5 anastomotic insufficiencies) and 7 SSI in the control group (5 superficial wound infections/dehiscences + 2 anastomotic insufficiencies). Thus, the concerns raised on discrepancies in our data do not result from errors but differences in definitions used.

## Allocated patients versus analysed patients

In accordance with the intention-to-treat principle, we collected data from the allocated groups, not from the subsequently analysed groups. In addition, to upholding the exchangeability between the intervention and control groups after randomisation, this minimises heterogeneity across the included studies, as decisions to exclude after randomisation may vary and are usually not random. Consequently, for the data collected from Bahlmann and colleagues,^2^ we used the number initially allocated (32 in each group) rather than the number subsequently analysed by the original authors. This approach minimised heterogeneity across the 75 studies included in our meta-analysis. In some of these studies, the reasons for excluding patients after allocation were unclear or varied. Thus, relying on the allocated numbers helps maintain consistency in standardised data reported within the meta-analysis.

We trust that this clarification resolves the concerns raised.

## Contributors

All authors were involved in the conceptualisation of the project, critically reviewed the draft manuscript for important intellectual content, approved the final version and agreed to accountability for all aspects of the work. Funding acquisition was done by MB. Project administration, methodology, data curation, investigation, visualisation, formal analysis and writing of the original draft were done by HJ and RH under the supervision of SJ, MB, and MH.

## Declaration of interests

RH reports receiving an institutional grant from Amsterdams Universiteitsfonds. DV reports receiving institutional grants or payments from Edwards Lifesciences and Philips Medical BV. MH reports receiving institutional grants, fees, royalties, or payments from ZonMW, European Society of Anaesthesiology and Intensive Care, PAION AG, IDD Pharma, Medical Developments, and Medirisk, and leadership or fiduciary role with Anesthesia & Analgesia, the Journal of Clinical Medicine, and Frontiers in Physiology. MB reports receiving institutional grants, payments, or honoraria from Dutch Association for Quality Funds Medical Specialists, Johnson & Johnson, Solventum, BD, Gore, Angiodynamics, KCI/3M, TELA Bio, Medtronic, and Smith & Nephew. All other authors declare no competing interests.

## References

[1] Jalalzadeh H., Hulskes R.H., Weenink R.P. (2024). Systematic review and meta-analysis of goal-directed haemodynamic therapy algorithms during surgery for the prevention of surgical site infection. eClinicalMedicine.

[2] Bahlmann H., Halldestam I., Nilsson L. (2019). Goal-directed therapy during transthoracic oesophageal resection does not improve outcome: randomised controlled trial. Eur J Anaesthesiol.

[3] Horan T.C., Gaynes R.P., Martone W.J., Jarvis W.R., Emori T.G. (1992). CDC definitions of nosocomial surgical site infections, 1992: a modification of CDC definitions of surgical wound infections. Infect Control Hosp Epidemiol.

